# Efficacy of acupuncture and its influence on the emotional network in adult insomnia patients: protocol for a randomized controlled clinical trial

**DOI:** 10.1186/s13063-021-05913-2

**Published:** 2022-01-04

**Authors:** Tongfei Jiang, Qi Zhang, Fang Yuan, Fan Zhang, Jing Guo

**Affiliations:** 1 Department of Acupuncture and Moxibustion, Beijing Hospital of Traditional Chinese Medicine, Capital Medical University, Beijing Key Laboratory of Acupuncture Neuromodulation, 23 Art Museum Backstreet, Dongcheng District, Beijing, 100010 China; 2 Graduate School, Beijing University of Chinese Medicine, Beijing, 100029 China; 3 Graduate School, Capital Medical University, Beijing, 100053 China

**Keywords:** Acupuncture, Insomnia disorder, Emotional network, Functional magnetic resonance imaging

## Abstract

**Introduction:**

Insomnia disorder (ID) is characterized by dissatisfaction with the quantity or quality of sleep and is often accompanied by negative emotions such as anxiety and depression. Patients with insomnia become trapped in a vicious circle of bad moods and poor sleep. Resting-state functional magnetic resonance imaging (r-fMRI) studies have shown abnormalities in emotion-related brain networks in patients with ID. And it has been proven that reducing negative emotions improves sleep quality. As a traditional alternative therapy, acupuncture has been demonstrated to be effective not only in improving sleep quality but also in stabilizing emotions; however, the mode of action needs to be further explored. Therefore, a clinical trial was designed to explore the effect of acupuncture in improving sleep and mood and to intuitively investigate the regulation of the emotional network using fMRI.

**Methods and analysis:**

A total of 60 participants with ID will be randomly allocated to a spirit-regulating group or a control group using non-effective acupoints acupuncture at a ratio of 1:1. All participants will receive 3 acupuncture treatment sessions per week for 4 weeks. In addition, 30 healthy individuals will be included in the healthy group. The primary outcome is the Pittsburgh Sleep Quality Index (PSQI). Secondary outcomes are the Hamilton Anxiety Scale (HAMA), the Hamilton Depression Scale (HAMD), the Hyperarousal Scale (HAS), and the Fatigue Scale-14 (FS-14), r-fMRI data, sleep diary, and actigraphy. The data will be collected prior to treatment, following treatment, and during the 12-week follow-up period; a sleep diary will be kept during the entire process.

**Ethics and dissemination:**

This protocol has been approved by the Research Ethical Committee of Beijing Hospital of Traditional Chinese Medicine (Bejing TCM Hospital). The results will be published in peer-reviewed journals or presented at academic conferences.

**Trial registration:**

Chinese Clinical Trials Register ChiCTR1800015282.

Protocol version: Version 1.0. Date: Dec.2020

## Background

Insomnia disorder (ID) is essentially characterized by difficulty falling asleep or maintaining sleep despite adequate opportunity and circumstances. The patients always show dissatisfaction with the quantity and/or quality of sleep and complain of impairments in daytime social functioning and work [1]. A survey found that more than half of questioned adults worldwide had sleep difficulties and 22.1% met the diagnostic criteria for insomnia disorder (DSM-4) [2]. In China, a meta-analysis suggests that the prevalence of ID has reached 15% [3].

ID is the second most prevalent mental illness worldwide and one of the leading causes of depression, anxiety, dementia, and other mental disorders. With the growing number of insomniacs, the incidence of psychological disorders is increasing [4, 5]. Persistent ID can be extremely burdensome and evoke anxiety, a lack of self-satisfaction, and even a fear of sleep. In turn, these negative emotions worsen insomnia, ultimately creating a vicious cycle of conflict [6]. A significant correlation between depression and ID has been revealed [7]; insomnia is a predictor of the onset of mental disorders [8], while individuals who have difficulties in emotional regulation are prone to ID [9].

R-fMRI indirectly reflects the level of human cortical activity according to changes in cerebral vascular blood oxygen supply. It has been suggested that there exists abnormal activity in multiple brain regions and networks in patients with ID, such as the default mode network (DMN), dorsal attention network, and sensory–motor network [10, 11].

Emotional reception and expression are mostly associated with the amygdala, medial prefrontal cortex (mPFC), insula, anterior cingulate cortex (ACC), and thalamus [12, 13], brain regions collectively referred to as the emotional network (EN) [12, 14]. Previous studies have shown that the left mPFC and insula are associated with sleep maintenance [15, 16]. Zhu et al. [17] found that the functional connectivity (FC) values of the left ACC and right insula are closely related to the severity of anxiety in ID patients. Another study suggested that the abnormal structure and function of the insula may negatively impact sleep and mood [18] and that the connection between the anterior insula and the left dorsolateral PFC is critical to antidepressant and anti-insomnia effects [19, 20]. Pang et al. [21] suggested that diminished mPFC activity is a neurobiological marker of cognitive impairment and chronic ID. Sanford et al. [22] also provided evidence that the mPFC plays an important role in the regulation of sleep and cognition. These studies suggest a strong relationship among insomnia, bad moods, and abnormal EN functional connectivity.

The main first-line treatments for ID are pharmacotherapy and cognitive behavioral therapy (CBT). Dominant anti-insomnia medicines include benzodiazepines, melatonin, and appetite-stimulating receptor antagonists [23], which are often accompanied by side effects such as “hangovers,” fatigue, and dependence. Some antidepressants are used in the treatment of insomnia, indicating that improving mood is beneficial for sleep regulation. CBT can also improve the poor mood of insomnia patients, but its clinical use is limited due to poor compliance [24].

As a traditional alternative therapy, acupuncture has been proven to be effective in insomnia according to evidence-based data [25–27]. A systematic evaluation showed that acupuncture can decrease the PSQI score, with a lower incidence of adverse events than that seen with anti-insomnia medicine [28, 29]. It has also been demonstrated that acupuncture can improve sleep quality, relieve daytime sleepiness [30], and decrease HAMA, HAMD-17, and Self-Rating Depression Scale (SDS) scores [31, 32]. This suggests that acupuncture can not only improve sleep quality but also relieve dysphoria.

According to traditional Chinese medicine, the method of spirit regulation is key to treating mental and emotional diseases with acupuncture. Spirit-regulating acupuncture has been proven to calm the mind, decrease hyperarousal and daytime fatigue, and adjust emotions [33].

Acupuncture may function by regulating brain activity in ID patients. Previous studies have revealed that acupuncture can decrease the excessive FC between the amygdala and hippocampus, posterior cingulate gyrus, lingual gyrus, and occipital lobe in insomniacs [11, 34]. Zhou et al. [35] found that electroacupuncture stimulation of the Shenmen and Sanyinjiao acupoints can activate the anterior ventral thalamic nucleus, caudate nucleus, shell nucleus, medial pallidum, and reticular nucleus of the thalamus in ID patients. Moreover, acupuncture at the Baihui acupoint for insomnia may function by activating signals in the hypothalamus and temporal lobe areas and adjusting signals in the frontal lobe areas to relieve anxiety and depression and improve sleep [36].

Hence, we hypothesize that spirit-regulating acupuncture will regulate activity within the EN, thus calming the mood and improving sleep quality. Therefore, we propose to investigate the effect of acupuncture on EN activity in ID patients with a view to elucidating the underlying central neural mechanism.

## Methods and analysis

### Study design

**Fig. 1:**
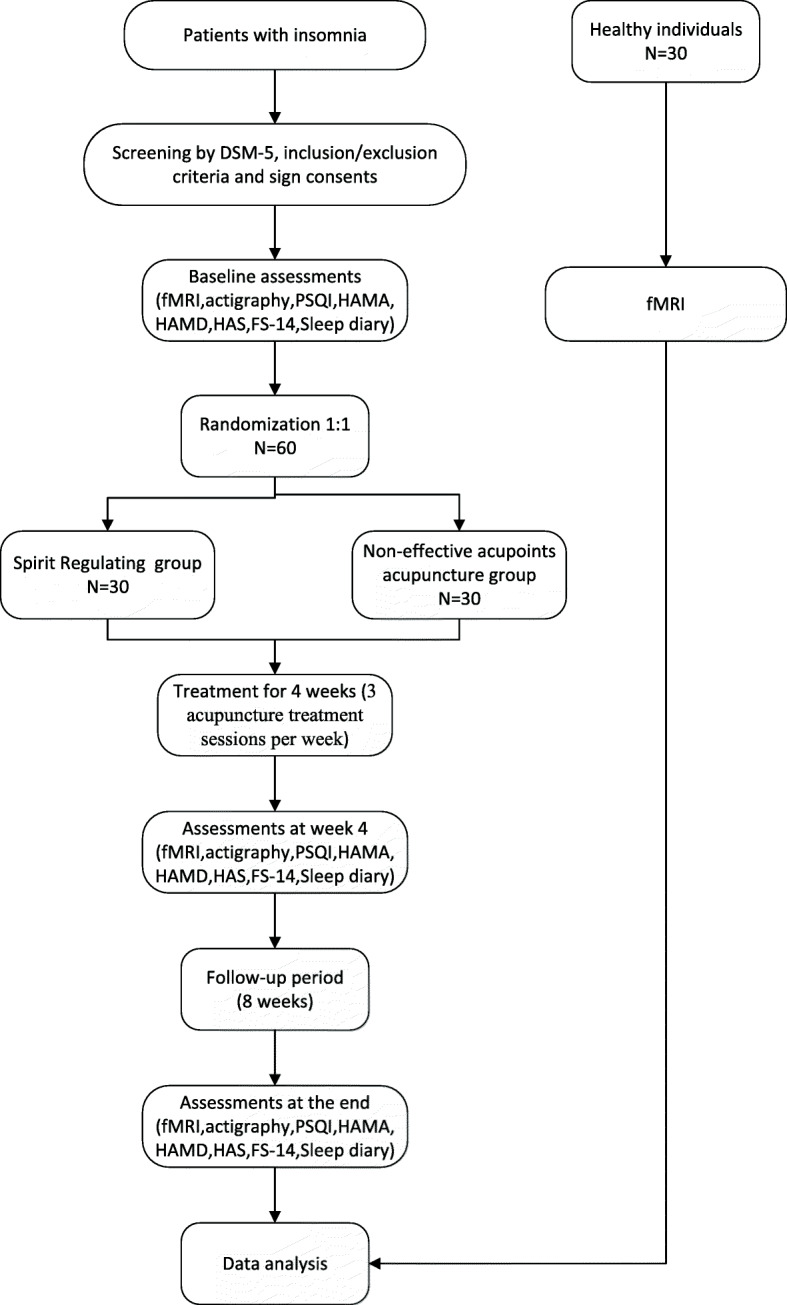
Study flowchart

This is a single-center, randomized, sham-controlled, observer- and patient-blinded trial involving two parallel groups using a 1:1 allocation ratio. Thirty age- and sex-matched healthy individuals who sleep well will be recruited as controls for the fMRI data analysis. A diagram of the trial design is provided in Fig. 1 and Table 1.

**Table 1:**
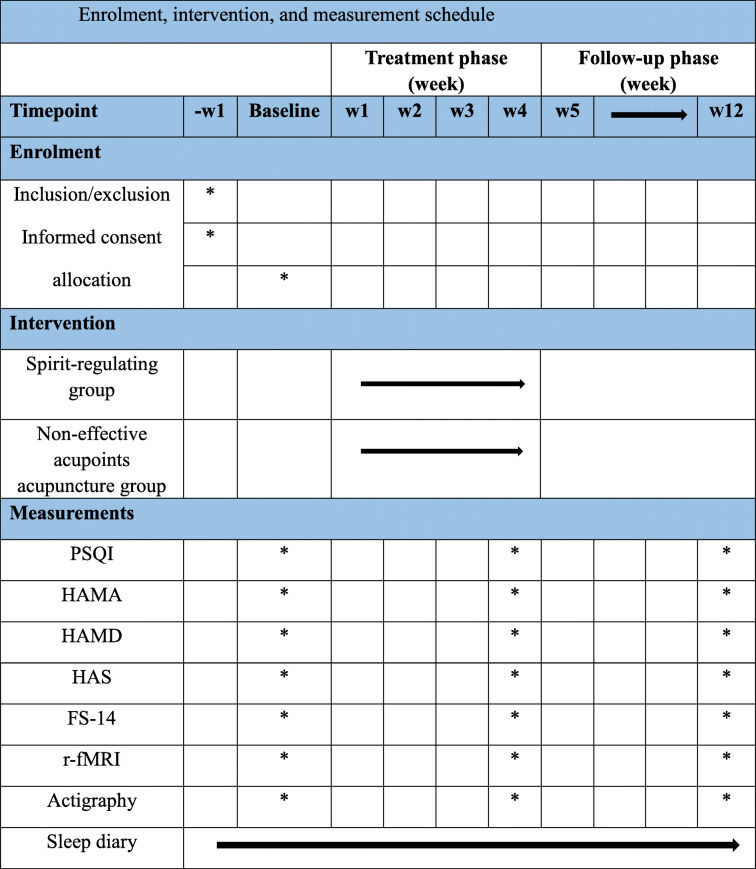
Enrolment, intervention, and measurement schedule

### Objective

To assess the efficacy of acupuncture in improving sleep quality and mood in ID patients and to investigate the changes in EN activity following spirit-regulating acupuncture treatment.

### Hypotheses

We hypothesize the following:

1. In comparison with the non-effective acupoints acupuncture group, the spirit-regulating group will display significantly relieved symptoms of insomnia, anxiety, and depression following treatment, as measured by valid scales.
2. In the spirit-regulating group, EN function will be modified based on fMRI examination as compared with the control group.

### Participants and recruitment

A total of 60 patients diagnosed with ID according to *the Diagnostic and Statistical Manual of Mental Disorders* (DSM-5) will be recruited from the outpatient Acupuncture Department of Beijing TCM Hospital or using advertisements via the internet or social media. The clinicians will be responsible for enrolling participants who are willing to undergo acupuncture treatment for ID. The assistant researchers will assess and record participant baseline statuses. After written informed consent has been obtained, eligible participants will be randomly allocated according to a random number table.

### Randomization and allocation concealment

Random numbers will be generated by the SAS statistical analysis system and sealed in an opaque envelope. According to the group code, the ID patients will be randomly assigned to one of the two groups and receive different treatments (spirit-regulating acupuncture or non-effective acupoints acupuncture). Random numbers will be assigned by telephone by an individual not involved in this study. Qi Zhang will generate the allocation sequence, Tongfei Jiang will enroll participants, and Jing Guo will assign participants to interventions and perform acupuncture operations

### Blinding

Owing to the characteristics of acupuncture, acupuncturists involved in this trial cannot be blinded to the assignments. The non-effective acupoints acupuncture design can however guarantee a good blinding effect for the participants. Assessors and statisticians involved in data collection and analysis will be blinded to the assignments.

## Participants

### Inclusion criteria

Patients who meet the diagnostic criteria for DSM-5 and the following requirements will be enrolled.

1. Aged 20–60 years.
2. Pittsburgh Sleepiness Scale (PSQI) score > 8.
3. Hamilton Depression Rating Scale (HAMD) score < 7.
4. Hamilton Anxiety Scale (HAMA) score < 14.
5. Hyperarousal Scale (HAS) score > 32.
6. Informed consent signed by the subject themself or an immediate family member.
7. Not taking anti-anxiety, anti-depressant, or sleep medications in the past month.

### Exclusion criteria

If any of the following criteria are met, patients will be excluded from the study.

1. Depression, anxiety, schizophrenia, or other serious mental illnesses.
2. Severe heart, brain, kidney, or liver disease.
3. Failure to cooperate with examination and treatment.
4. Apnea syndrome.
5. Pregnant and lactating women.
6. Claustrophobia or other contraindications to MRI examination.
7. A definite lesion on MRI or grossly asymmetrical anatomy of the head.

## Intervention

All acupuncture procedures will be performed by one acupuncturist who is a licensed TCM practitioner. The frequency and duration of treatment in the two groups will be the same. Subjects were prohibited from receiving any sleep-assisting drugs or physical therapy during the course of the experiment.

### Spirit-regulating group

The Baihui (DU20), Shenting (DU24), Sishencong (EX-HN1), Shenmen (HT7), Benshen (**GB**13), Neiguan (PC6), and Sanyinjiao (SP6) acupoints will be used. The positioning standard refers to the *National Standard for Acupuncture and Moxibustion Meridian Point Positioning* promulgated by the National Standard GB12346-90 of China. All patients will lie on a bed in the supine position, and acupoints will be sterilized using 75% alcohol on a cotton swab. The acupuncturist will insert disposable stainless-steel needles (Huatuo, Suzhou, China; 0.25 × 40 mm) into the acupuncture points. For DU20, DU24, EX-HN1, and **GB**13, the needle will be inserted 10 mm horizontally; for HT7 and PC6, 5 mm vertically; and for SP6, 10 mm vertically, accompanied in all cases by twisting of the needle to produce a sensation of Deqi (the key to effective acupuncture). Participants will undergo a 30-min treatment per session. There will be 3 acupuncture treatment sessions per week for 4 consecutive weeks, giving a total of 12 sessions.

### Non-effective acupoints acupuncture group

**Fig. 2:**
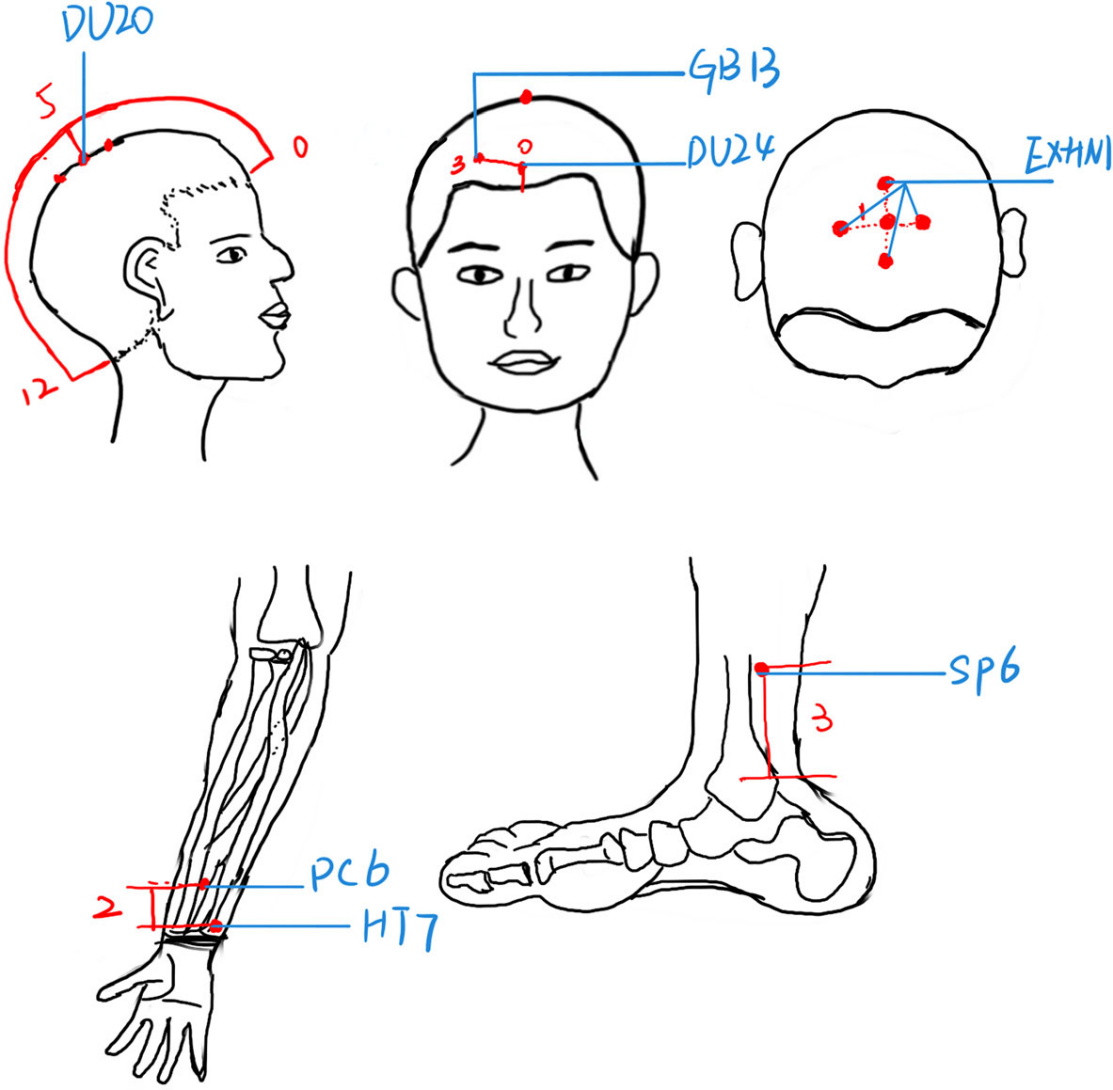
Locations of the acupoints in the spirit-regulating group

**Fig. 3:**
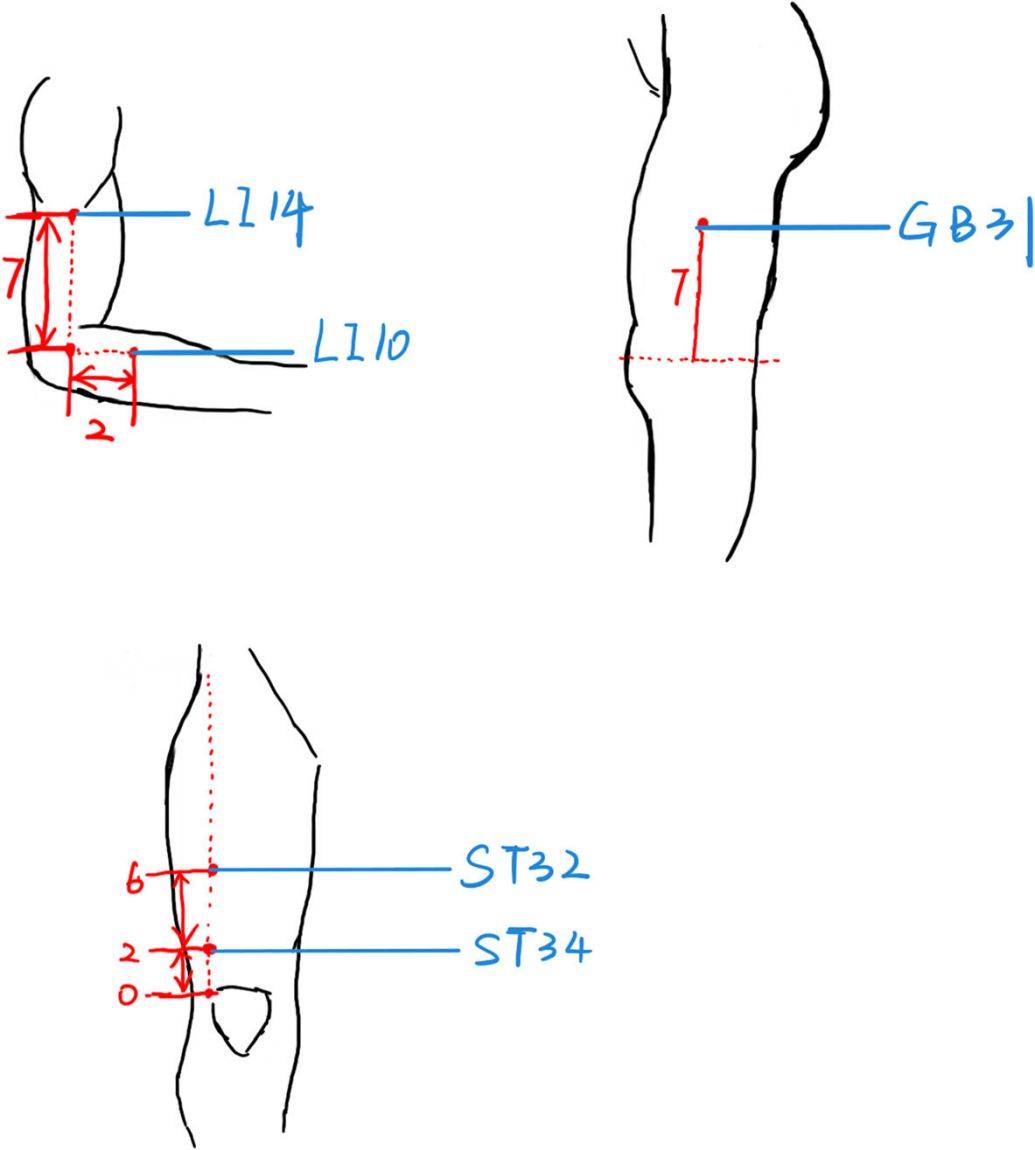
Locations of the acupoints in the non-effective acupoints acupuncture group

In the non-effective acupoints acupuncture group, the Binao (LI14), Shousanli (LI10), Fengshi (GB31), Futu (ST32), and Liangqiu (ST34) acupoints will be subjected to vertical insertion of 1–2 mm, avoiding the sensation of Deqi. Acupuncture in non-effective acupoints on the superficial surface of the skin can achieve a blinded effect, and the patients will be convinced that effective acupuncture has been performed. Participants will also undergo a 30-min treatment per session. The frequency of treatment will be the same as that in the spirit-regulating group. If a subject suffers a serious adverse injury (e.g., severe local infections, injury to vital vessels, stabbing the organs and nerves) from acupuncture during the experiment, the experiment may be terminated and the subject may voluntarily withdraw (Figs. 2 and 3).

## Outcome

### Primary outcome

The primary outcome measure will be the change in the PSQI score at week 4 as compared with that at baseline. The PSQI is the most commonly used indicator for the evaluation of sleep quality in insomniacs with respect to sleep duration, efficiency, depth, and abnormal sleep sensations. The higher the score, the worse the sleep. Reference of PSQI scores: 0~5 sleep quality is very good; 6~10 Sleep quality is okay; 11~15 average sleep quality; 16~21 sleep quality is very poor [37].

### Secondary outcomes

#### The Hamilton Anxiety Scale (HAMA)

The HAMA is a reliable and valid anxiety evaluation questionnaire. Anxiety is the main adverse emotion in most ID patients, and it has been proven that insomnia is highly comorbid with anxiety [38]. The HAMA is composed of 14 questions and is used as an efficacy index to evaluate the severity of anxiety. The higher the score, the more anxious the patient.

The Hamilton Depression Scale (HAMD)

The HAMD is composed of 17 questions and is used to evaluate the severity of depressed mood. The higher the score, the more depressed the patient. In our trial, patients with mild depression symptoms (HAMD scores less than 7) will be selected for inclusion.

#### The Hyperarousal Scale (HAS)

Insomniacs have higher levels of cortical arousal. A total of 26 self-assessment items are included in the HAS, which is used to assess patient hyperarousal status. The higher the score, the higher the level of cortical arousal.

#### The Fatigue Scale (FS-14)

Patients with insomnia are more likely to experience fatigue and weakness. The FS-14, containing two parts regarding physical and mental fatigue, shows patient physical and psychological fatigue statuses. The higher the score, the greater the fatigue.

#### The sleep diary

Patients will be asked to fill out the sleep diary from 1 week prior to the start of acupuncture treatment until the end of the follow-up period. This diary includes the time of falling asleep and waking up, sleep quality, and factors that affected sleep.

#### Actigraphy

Participants will be requested to wear an actigraph unit (MTI Health Services Company, Pensacola, FL, USA) on the left wrist. Actigraphy is an objective indicator that reflects sleep time and quality in ID patients. We will collect actigraphy data for three 1-week periods (before the intervention, at the end of the intervention period, and at the end of the follow-up period).

All fMRI data will be acquired at the Imaging Department of Beijing TCM Hospital by the same skilled and professional technician using a Siemens TRio 3.0 Tesla MRI scanner. A professionally trained member of the medical staff will explain the r-fMRI precautions and procedure to the participants 30 min prior to examination. All participants will undergo whole-brain conventional structural imaging and fMRI scans. Conventional r-fMRI scan parameters are as follows: voxel size = 3.0 × 3.0 × 3.0 mm^3^, 32 axial slices, thickness = 3.0 mm, repetition time = 2000 ms, echo time = 30 ms, flip angle = 90°, field of view = 220 × 220 mm^2^, and matrix size = 94 × 94. During acquisition of fMRI data, all participants will be asked to lie on the MRI bed, stay relaxed, quiet, and still but awake with open eyes, and to try not to think about anything. Each participant will be examined three times during the entire process: prior to treatment, following treatment, and after the follow-up period.

We will select brain areas within the emotional network (EN): the amygdala, medial prefrontal cortex (mPFC), anterior cingulate cortex (ACC), insula, thalamus, and hypothalamus as seed points (Talairach coordinates). The mean time-series of these seed points will be extracted, and voxel FC analysis will be performed for each seed point.

### Safety assessment

Acupuncture-related adverse events include severe sharp pain (visual analog scale ≥ 7), hematoma or bleeding at the site of needle insertion, nausea, and cold sweats during treatment. If the subjects experience other needle-related discomforts, it should be recorded and will be assessed throughout the study in both groups. There is no anticipated harm and compensation for trial participation. If the subjects need it, we will continue to give sleep health instructions for post-trial care.

### Data management and monitoring

To ensure standardization, clinical training sessions will be held for each investigator prior to the start of the trial. The training will include proper application of the random number table, making diagnoses, understanding inclusion and exclusion criteria, and completing the case report forms. Importantly, acupuncture will be performed by the same acupuncturist according to standard acupuncture point positioning. In this way, we can improve inter-observer consistency among researchers and ensure the reliability of clinical research findings. Data recording will be required to be timely, accurate, complete, and standardized. The information collector collects the personal information and experimental date of the participants, verifying that data collected during the course of the research will be kept strictly confidential and only accessed by members of the trial team (or individuals from the Sponsor organization or center sites were relevant to the trial). Participants will be allocated an individual trial identification number and their details will be stored on a secure database. The statistician is only responsible for data analysis and does not know the source of the information, by these ways to keep it safe and prevent leakages. The sponsor of this trial will conduct an interim analysis in due course and will have access to the clinical data. Anonymous trial data could be shared with other researchers to international prospective meta-analyses. The data of this study will be supervised by the Research Department of Beijing TCM Hospital. Auditors will monitor the progress of the experiment every 14 days and record the progress of the trial, the process that will be independent from investigators and sponsor. Any data required to support the protocol can be supplied on request.

In addition, fMRI scanning will be performed using the same scanner at Beijing TCM Hospital. All subjects will be scanned in a unified state, including open-eyed, avoiding thinking, remaining motionless, and staying awake. To obtain better compliance, the following measures will be applied: (1) following the voluntary principle, which the investigator will fully introduce the purpose of the study, the process and the possible effect of acupuncture treatment, and the subjects will voluntarily enroll and they have the right to drop out at any time after agreeing to participate in the study without any discrimination or retaliation; (2) signing a patient informed consent form; (3) making an effort to establish a good relationship between the doctor and the patients; and (4) recording their contact details for follow-up.

### Protocol amendments

The study plan of this project has been registered with approval number ChiCTR1800015282. If any changes need to be made for the protocol, we will first notify the sponsor and funder, then the principal investigator (PI) will notify the centers, and that a copy of the revised protocol will be sent to the PI to add to the Investigator Site File. Any deviations from the protocol will be fully documented using the breach report form. The PI of this study will update the protocol in the clinical trial registry.

## Statistical methods

### Sample size

In this trial, PSQI results are the primary outcome. In our previous pilot study [39], the PSQI score significantly decreased by 4.43±3.60 in the acupuncture group and by 1.30±2.58 in the control group. Using a *t* test of two independent samples with uneven variance in PASS for calculation, the withdrawal rate is 20% to ensure that the results are statistically different (*α* = 0.05, 1-β = 0.9); therefore, each group requires 29 subjects. To improve the reliability of the trial, we will recruit a total of 60 ID patients, with 30 in each group.

### Clinical data analysis

Data from the trial will be evaluated using the SPSS software V21.0 (IBM SPSS Statistics, IBM Corp, Somers, NY, USA). Apply the multiple interpolation method to complete some of the missing data. All demographic and baseline characteristics will be analyzed using different approaches. A chi-square test will be used to compare the demographic information between the two groups. Differences between group means will be assessed using repeated-measures analysis of variance. The main objective is to assess the difference in change in PSQI score between the groups from baseline to week 4; an independent samples *t* test will be used for comparison. For secondary outcome data such as HAMA, HAMD, HAS, and FS-14, an independent samples *t* test (*p* < 0.05) will be used to compare differences between the two groups, and a paired *t* test (*p* < 0.05) will be used to compare patients in the same group before and after treatment. Subjects who participated in the randomized group, whether they received treatment in that group or not, were eventually included in the assigned group for statistical analysis of efficacy, and this experiment followed ITT (intention-to-treat analysis) principles [40].

### Functional MRI data analysis

Data preprocessing and calculations of FC will be performed using DPABI [41] in MATLAB_R2018a (Mathworks, Inc., Natick, MA, USA). The raw data will be preprocessed as follows: format conversion, removal of the first 10 time points, slice timing, realignment, normalization, smoothing, covariates regressor application, and filtering.

After completion of data preprocessing, the next step will be FC analysis. The amygdala, mPFC, ACC, insula, thalamus, and hypothalamus will be selected as seed points (Talairach coordinates), and the mean time-series of these seed points will be extracted, and voxel FC analysis will be performed for each seed point. The Pearson correlation coefficient of the average time-series between each brain region within the EN and this seed point will be calculated, and its value will be used as the FC strength. Regions with statistically significant strength of FC with seed sites will be considered functionally relevant to the seed sites. To follow normal distribution, the resulting correlation coefficients will need to be converted to *z* values using Fisher’s *r*-*z* transformation. Sociodemographic information, including age, years of education, PSQI score, HAMA score, and HAMD score, will be used to assess the differences between the two groups using a two-sample *t* test. The difference in gender between the two groups will be analyzed using a chi-square test. For MRI data, a two-sample *t*-test will be used to statistically derive FC values for the spirit-regulating group versus the non-effective acupoints acupuncture group. The threshold for correction of cluster levels will be set at *P* < 0.05 and considered statistically significant. Multiple corrections will be performed using Alphasim. Statistical analysis of data will be performed using the MATLAB, DPABI, and SPSS software. A paired *t* test will be used for intra-group comparisons; an independent sample *t* test will be used for inter-group comparisons; and Pearson’s correlation analysis will be used to describe the relationship between imaging data and scale scores.

## Discussion

To the best of our knowledge, this is the first clinical trial to explore the influence of acupuncture on emotional network (EN) function. By analyzing the correlation between EN functional connectivity and clinical evaluation, we expect to reveal the neurological mechanism of spirit-regulating acupuncture treatment for ID.

This is a randomized, observer- and patient-blinded, controlled clinical trial sponsored and financially supported by the National Nature Science Foundation of China. According to traditional Chinese medicine, poor sleep quality is closely related to negative emotions such as depression and anxiety. Previous research has shown that acupuncture is effective in improving sleep and mind-tranquilizing in ID patients at the same time.

We hypothesize that acupuncture may relieve symptoms of depression and anxiety. by adjusting the emotional brain regions in ID patients, resulting in an improvement in sleep. The effect of acupuncture on emotional conditions and the underlying neural mechanism have been sparsely studied; thus, we designed the trial to identify the relationship between the effect of acupuncture in regulating emotions and the regulation of EN function.

In our trial, the subjective and objective outcomes including PSQI, HAMA, HAMD, HAS, FS-14, actigraphy data, and a sleep diary will be assessed. In this way, the effect of acupuncture will be evaluated from multiple perspectives. Moreover, we have established an 8-week follow-up to observe the sustained effects of acupuncture.

In addition to neurobehavioral assessment, fMRI technology has been selected to reveal the neural mechanism of acupuncture in relieving insomnia and adverse emotion. It has been shown that there is abnormal FC within the EN in insomniacs [42, 43] and acupuncture may function to regulate the activity of emotion-related brain areas in ID patients [34–36]. In our study, FC analysis will be used to explore the effect of acupuncture on EN function in ID patients.

Inevitably, there also exist limitations to our trial. Firstly, the method of superficial and minimal needling at acupoints unrelated to insomnia has been chosen as a control; however, the method inevitably has some non-specific physiological effects. A non-penetrating method would be more suitable but it is difficult to conduct for subject acquaintance of acupuncture. A perfect comparison for real acupuncture with fewer physiological effects should be developed in the future. Secondly, according to our previous clinical observations, participants in the non-effective acupoints acupuncture group may have poor compliance as compared with those in the spirit-regulating group; hence, we will maintain close and friendly contact with all patients to minimize the dropout rate. Thirdly, the sample size for this trial is 30 patients per group, which may be underpowered for confirmation of the study hypothesis. In the future, a study will be designed with a greater sample size to give more confidence to the data.

In conclusion, the results of our trial are expected to demonstrate modulation of the EN by spirit-regulating acupuncture, which may reveal the potential central mechanism of acupuncture in the treatment of ID.

### Trial status

This trial is currently in the recruitment phase.

## Data Availability

All study-related data will be stored securely at the Beijing TCM Hospital. The datasets analyzed during the present study are available from the corresponding author for future secondary analysis.

## Abbreviations

ID: Insomnia disorder
PSQI: Pittsburgh sleep quality index
FS-14: Fatigue scale-14
HAMA: Hamilton anxiety scale
HAMD: Hamilton depression scale
HAS: Hyperarousal scale
r-fMRI: Resting-state functional magnetic resonance imaging
EN: Emotional network
FC: Functional connectivity
